# Supplementary material for: Effects of Cancer Presence and Therapy on the Platelet Proteome
Source: Int J Mol Sci. 2021 Jul 30;22(15):8236. doi: 10.3390/ijms22158236 (PMC8347210; doi:10.3390/ijms22158236)
Supplement: Supplementary file 1 [file ijms-22-08236-s001.zip › ijms-1266033-supplementary/ijms-1266033-supplementary Figures.pdf]

Supplemental figure S1: Gel electrophoresis and Coomassie staining of platelet samples from patients with cancer (n=9) and healthy controls (n=10).

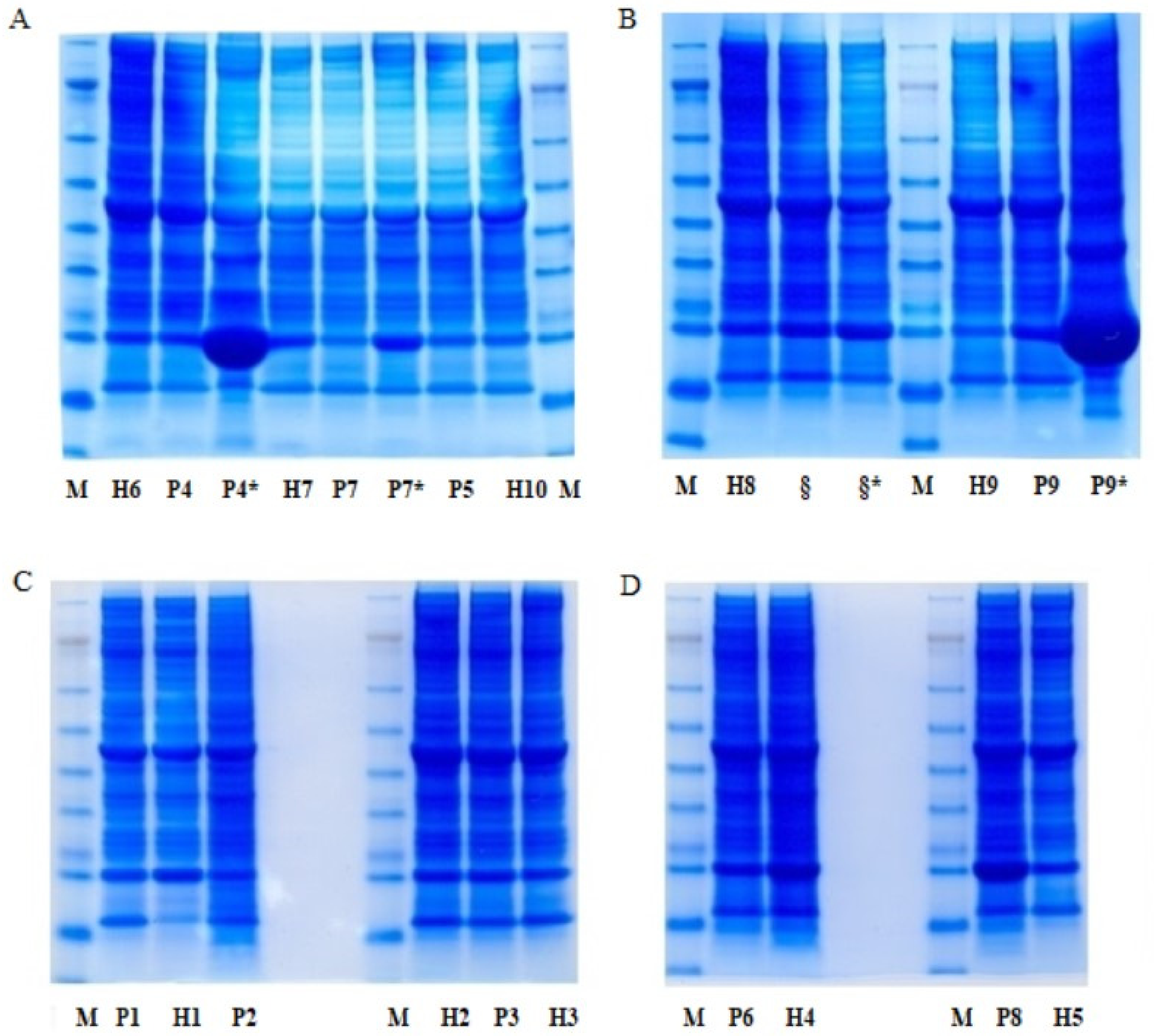

M=marker, H=Healthy, P=patient, *=after start of treatment, §Patient was excluded from further analysis due to meeting of exclusion criteria.

Supplemental figure S2:

Heat map and unsupervised cluster analysis and principal component analysis using protein expression data from platelets of patients with cancer (n=9) and healthy controls (n=10).

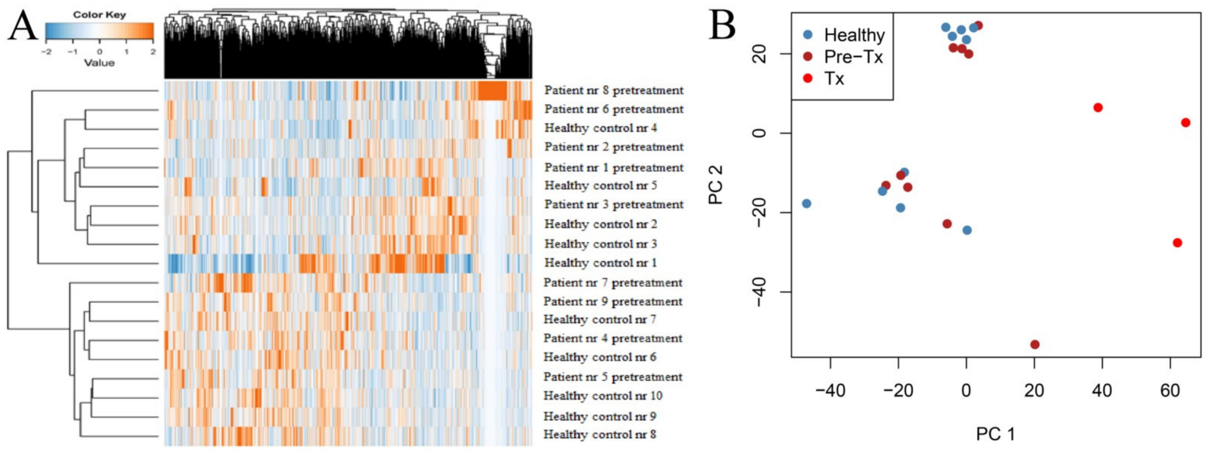

A)Heat map and unsupervised cluster analysis using protein expression data from platelets of patients with cancer (n=9) before treatment and healthy controls (n=10). The unsupervised cluster analysis does not show a clear separation of samples.

B)Principal component analysis using protein expression data from platelets of patients with cancer (n=9) before treatment and after start of treatment (n=3) and healthy controls (n=10). This analysis does not show a clear separation of the samples of patients before treatment and of the healthy volunteers.

Supplemental figure S3:

Protein-protein networks of platelet proteins, in paired patient samples, with a higher abundance before compared to after initiation of antitumor treatment.

($p < 0.05$, fold change ≥ 5). The networks were generated using default settings in String and visualized using Cytoscape.

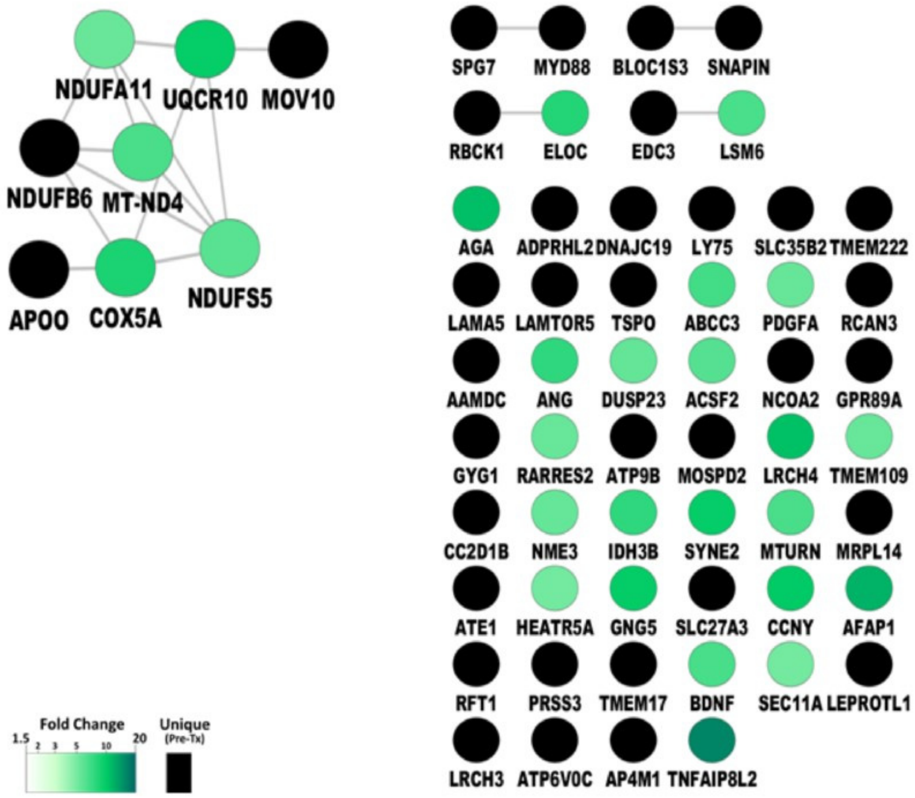
NDUFA11
UQCR10
MOV10
NDUFB6
MT-ND4
APOO
COX5A
NDUFS5
Fold Change
1.5
2
3
5
10
20
Unique
(Pre-Tx)
SPG7
MYD88
BLOC1S3
SNAPIN
RBCK1
ELOC
EDC3
LSM6
AGA
ADPRHL2
DNAJC19
LY75
SLC35B2
TMEM222
LAMA5
LAMTOR5
TSPO
ABCC3
PDGFA
RCAN3
AAMDC
ANG
DUSP23
ACSF2
NCOA2
GPR89A
GYG1
RARRES2
ATP9B
MOSPD2
LRCH4
TMEM109
CC2D1B
NME3
IDH3B
SYNE2
MTURN
MRPL14
ATE1
HEATR5A
GNG5
SLC27A3
CCNY
AFAP1
RFT1
PRSS3
TMEM17
BDNF
SEC11A
LEPROTL1
LRCH3
ATP6V0C
AP4M1
TNFAIP8L2
