# Supplementary material for: Effects of Cancer Presence and Therapy on the Platelet Proteome
Source: Int J Mol Sci. 2021 Jul 30;22(15):8236. doi: 10.3390/ijms22158236 (PMC8347210; doi:10.3390/ijms22158236)
Supplement: Supplementary file 1 [file ijms-22-08236-s001.zip › ijms-1266033-supplementary/Table legends.pdf]

Table legends:

Supplemental table S1:

List of all 4200 database entries and the linked to 4059 unique proteins in platelets.

Supplemental table S2:

List of 118 proteins which were expressed significantly different in platelets from cancer patients compared to healthy controls.

Supplemental table S3:

List of 50 proteins which were at least 1.5-fold higher expressed in platelets from patients with cancer compared to healthy individuals.

Supplemental table S4:

List of 36 proteins which were at least 1.5-fold higher expressed in platelets from healthy controls compared to cancer patients.

Supplemental table S5:

Upregulated proteins in platelets after antitumor treatment.

Supplemental table S6:

Downregulated proteins in platelets after antitumor treatment.

Supplemental table S7:

Proteins in platelets uniquely identified after start antitumor treatment.

Supplemental table S8:

Proteins in platelets only identified before start of antitumor treatment
